# Inhibition of Caspase-8 does not protect from alcohol-induced liver apoptosis but alleviates alcoholic hepatic steatosis in mice

**DOI:** 10.1038/cddis.2017.532

**Published:** 2017-10-26

**Authors:** Fengjie Hao, Francisco Javier Cubero, Pierluigi Ramadori, Lijun Liao, Ute Haas, Daniela Lambertz, Roland Sonntag, Jörg- Martin Bangen, Nikolaus Gassler, Mareike Hoss, Konrad L Streetz, Johanna Reissing, Henning W Zimmermann, Christian Trautwein, Christian Liedtke, Yulia A Nevzorova

**Affiliations:** 1 Department of Internal Medicine III, University Hospital RWTH, Aachen, Germany; 2 Department of Immunology, Complutense University School of Medicine, Madrid, Spain; 3 12 de Octubre Health Research Institute (imas12), Madrid, Spain; 4 Institute of Pathology, Klinikum Braunschweig, Braunschweig, Germany; 5 Electron Microscopic Facility, Medical Faculty, University Hospital RWTH Aachen, Germany; 6 Department of Animal Physiology II, Complutense University School of Biology, Madrid, Spain

## Abstract

Hepatic apoptosis is involved in the progression of alcoholic liver disease (ALD). *Caspase-8*, the apical initiator in death receptor-mediated apoptosis, has been implicated in acute liver injury and in non-alcoholic steatohepatitis. However, the relevance of *Caspase-8* in the pathogenesis of ALD remains unclear. In the present study, we investigated the impact of *Caspase-8* in human and murine alcohol-induced apoptosis and in ALD. We investigated human samples from ALD patients, primary mouse hepatocytes, and hepatocyte-specific *Caspase-8* knockout (Casp8^Δhepa^) mice in acute and chronic models of ethanol (EtOH) administration. Caspase-8 activation was detected in liver biopsies from ALD patients, as well as in livers of wild-type (WT) mice after chronic ethanol feeding for 8 weeks using the Lieber-DeCarli model. Lack of *Caspase-8* expression in Casp8^Δhepa^ animals failed to prevent alcohol-induced liver damage and apoptosis. Instead, inhibition of Caspase-8 shifted the ethanol-induced death signals towards pronounced activation of the intrinsic, mitochondria-dependent apoptosis pathway in Casp8^Δhepa^ livers involving enhanced release of cytochrome c, stronger *Caspase-9* activation and specific morphological changes of mitochondria. *In vitro* and *in vivo* intervention using a pan-caspase inhibitor markedly attenuated alcohol-induced hepatocyte damage in a Caspase-8-independent manner. Surprisingly, EtOH-fed Casp8^Δhepa^ mice displayed significantly attenuated steatosis and reduced hepatic triglyceride and free fatty acids content. Caspase-8 is dispensable for alcohol-induced apoptosis, but plays an unexpected role for alcohol-dependent fat metabolism. We provide evidence that simultaneous inhibition of extrinsic and intrinsic apoptosis signaling using pan-caspase inhibitors *in vivo* might be an optimal approach to treat alcohol-induced liver injury.

Excessive alcohol consumption is the primary cause of chronic liver disease and liver-related mortality in Western countries. According to the report of the World Health Organization (WHO), approximately 50% of all global deaths in 2012 from liver cirrhosis were attributable to alcohol. Thus, the socioeconomic impact of alcoholic liver disease (ALD) is extraordinary high. However, up to now the cellular mechanisms involved in ALD remain poorly understood and specific treatment options are still lacking.^1^

Recent studies have suggested that the pathogenesis of ALD is associated with hepatocyte apoptosis, as it was found in patients with alcoholic hepatitis and correlated with disease severity.^2^ Thus it is tempting to hypothesize that early inhibition of the apoptotic pathways may prevent ALD progression. However, for the design of effective anti-apoptotic drugs, an in-depth knowledge of the general and specific mechanism leading to apoptosis in ALD is needed.

Apoptosis is a highly regulated and genetically controlled type of cell death, which can basically be executed via two different molecular pathways: the death receptor-mediated extrinsic pathway and the mitochondria-dependent intrinsic pathway. Because of the high level of death receptor expression in hepatocytes, the liver is predominantly predisposed to extrinsic apoptosis. Chronic alcohol consumption is associated with increased secretion of several inflammatory cytokines, such as TNFα and FasL.^2, 3, 4, 5^

Cytokine binding and stimulation of the associated death receptors (FasR and TNF-R1) subsequently triggers an apoptotic program that includes the induction of a caspase cascade, which is initiated by activation of *Caspase-8*. Subsequently, *Caspase-8* initiates effector caspases 3, 6, and 7, eventually leading to characteristic apoptotic cell death.^6, 7^

Another mechanism proposed to explain alcohol-induced hepatocyte apoptosis is the induction of cytochrome P450 isoenzyme 2E1 (CYP2E1) and the generation of reactive oxygen species (ROS). ROS trigger the intrinsic pathway via activation of pro-apoptotic members of the Bcl-2 family, which oligomerise on the outer mitochondrial membrane and cause mitochondrial dysfunction. Following its release from the mitochondria, cytochrome c recruits and activates the initiator *Caspase-9*, which in turn, initiates effector caspases (i.e., caspases 3, 6, 7) responsible for the degradation of cellular substrates.^2, 8^

Taken together, the end-point of both the intrinsic and the extrinsic pathways is activation of effector caspases and endonucleases that ultimately degrade the cellular constituents. However whether alcohol-induced apoptosis requires extrinsic or intrinsic signaling pathways or both remains unclear.

We recently generated and characterized genetically modified mice with deletion of *Caspase-8* specifically in hepatocytes (Casp8^Δhepa^).^9^ We provided evidence that hepatocyte-specific deletion of *Caspase-8* protected mice from Fas- or LPS-induced apoptosis. However, we also found that lack of *Caspase-8* may sensitize hepatocytes for necroptotic cell death in a strong inflammatory environment such as Concanavalin A treatment. Accordingly, the outcome of *Caspase-8* inhibition in ALD could hardly be predicted.

Therefore, in the present study we investigated the role of *Caspase-8* in hepatocytes for EtOH-induced liver damage. Our findings expand the current knowledge on the pathomechanism of ALD. Specifically, we demonstrate that *Caspase-8* is a crucial key driver of hepatic steatosis but absolutely dispensable for alcohol-induced cell death, due to compensatory crosstalks between the major apoptotic-inducing extrinsic and intrinsic pathways.

## Results

### Ethanol induces hepatic activation of Caspase-8 in human and murine ALD

**Figure 1:**
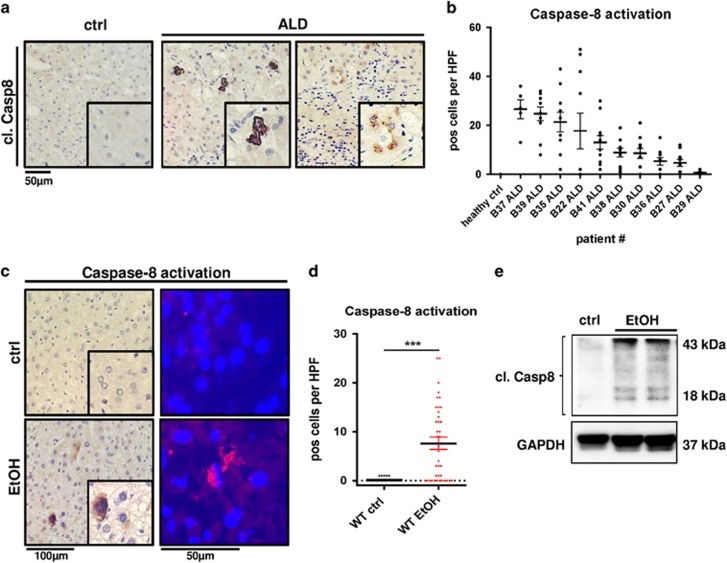
Caspase-8 is activated in human and murine ALD. (**a**) *In situ* Caspase-8 activation (brown) determined via immunohistochemistry in representative liver sections from patients with ALD and healthy controls (ctrl). Cells with activation of *Caspase-8* are highlighted by enlarged views. (**b**) Quantification of *Caspase-8*-positive cells in stained liver sections from individual patients. Ten randomly selected × 400 high power fields (HPF) per sample were analyzed and the number of *Caspase-8-*positive hepatocytes in each view field is indicated. (**c–e**) WT mice were fed isocaloric control diet (ctrl) or EtOH-containing Lieber-DeCarli diet for 8 weeks. (**c**) Left: Immunostaining for activated *Caspase-8* (brown) in paraffin sections of WT liver. Right: Immunofluorescence staining for activated *Caspase-8* expression (red) in frozen livers sections. (**d**) Quantification of *Caspase-8*-positive cells per × 400 HPF in paraffin sections. (**e**) Immunoblot analysis of *Caspase-8* protein expression and activation. Activation is indicated by cleavage products of 43 and 18 kDa. GAPDH was used as loading control. ***P<0.001

We first aimed to determine the relevance of *Caspase-8* in human ALD. Liver biopsy samples from patients with clinically and histologically proven ALD (Supplementary Table 1) were immunostained against cleaved (i.e., activated) *Caspase-8* and quantified. Samples from human healthy control specimens did not reveal *Caspase-*8 activation. In contrast, we found pronounced activation of *Caspase-8* in livers from ALD patients predominantly in hepatocytes (Figure 1a and b). Isotype controls were performed to exclude non-specific staining (Supplementary Figure 1a).

Next, we investigated the relevance of *Caspase-8* for the progression of ALD in mice. To this end we subjected wild-type (WT) mice to an EtOH-containing Lieber-DeCarli or an isocaloric control diet in order to induce chronic alcoholic liver damage. In agreement with our findings in human samples, immunohistochemical (IHC) and immunofluorescence (IF) stainings of murine liver sections showed that *Caspase-*8 was significantly activated after 8 weeks of alcohol feeding, and localized in the cytoplasm of hepatocytes (Figures 1c and d,Supplementary Figure 1b). Protein analysis confirmed enhanced activation of *Caspase-8* in alcohol-fed WT livers (Figure 1e). Altogether our findings suggest that *Caspase-8* activation is characteristic of murine and human ALD.

### Depletion of Caspase-8 does not prevent alcohol-driven liver injury in mice

To further determine the impact of *Caspase-8* in experimental ALD, we challenged Casp8^Δhepa^ mice with Lieber-DeCarli diet. The efficiency of *Caspase-8* deletion was confirmed by PCR and immunoblots in primary hepatocytes (Supplementary Figures 2a and b).

**Figure 2:**
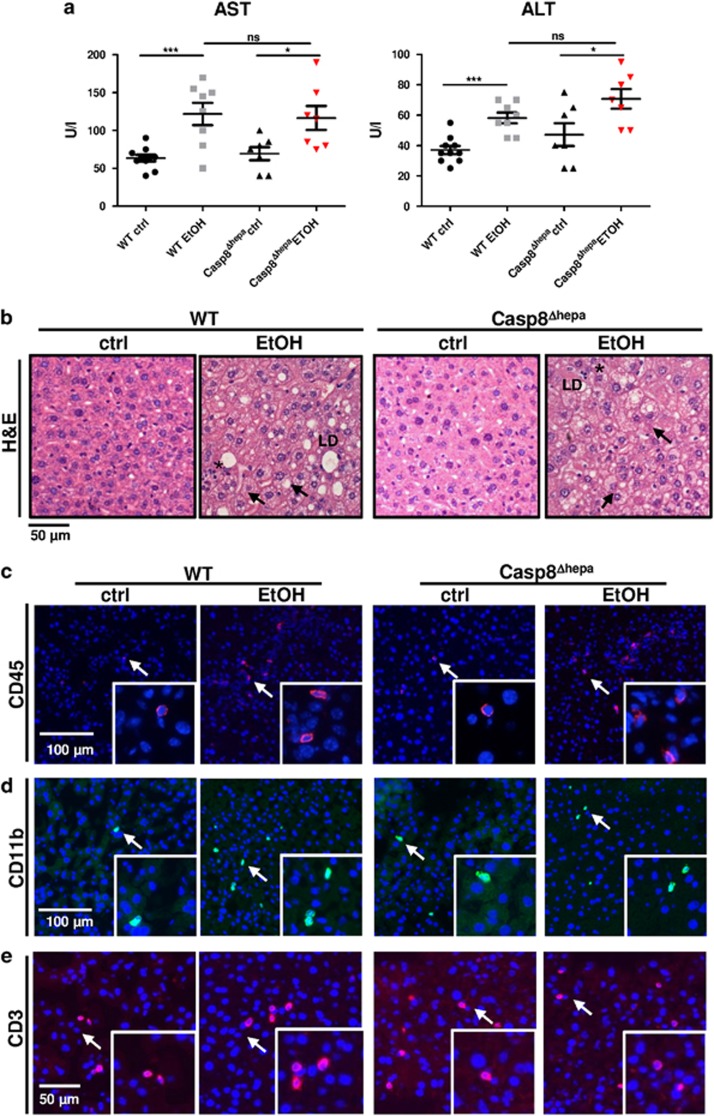
Ablation of *Caspase-8* does not prevent alcoholic liver injury in mice. WT (*n*=8–10) and Casp8^Δhepa^ mice (*n*=7) were fed with isocaloric (ctrl) or Lieber-DeCarli (EtOH) diet for 8 weeks. (**a**) Serum AST and ALT activities after 8 weeks of feeding. (**b**) Representative H&E staining of liver sections from each treatment group. Arrows: ballooned enlarged hepatocytes with rarefied cytoplasm; Black asterisk: polymorphonuclear neutrophils; LD: lipid droplets. (**c–e**) Determination of pro-inflammatory effects through immunofluorescence staining for **(c)** CD45 (red, arrows), (**d**) CD11b (green, arrows) and (**e**) CD3 (red, arrows). Total cells are counterstained with DAPI (blue). ***P<0.001; *P<0.05; n.s.: not significant

Mice receiving Lieber-DeCarli diet for 8 weeks modestly but significantly increased basal plasma levels of transaminases (AST, ALT) indicating cellular liver injury. Surprisingly, ethanol-induced increase in transaminase activities was not prevented by *Caspase-8* inactivation (Figure 2a).

Histological liver examination did not reveal major differences between WT and Casp8^Δhepa^ animals (Figure 2b). Liver sections in both groups revealed typical histological appearance of EtOH-induced cellular damage including hepatocytes ballooning, edema and cytoplasm rarefaction with intra-cytoplasmic vague microvesicular lipid droplets and residual granular materials. We did not observe confluent necrosis; however, accumulation of polymorphonuclear neutrophils was often associated with individual cell loss. Of note, WT livers more frequently exhibited signs of macrosteatosis involving large fat inclusions, occupying the whole cell and displacing the cytoplasm and nuclei.

Next, we investigated the relevance of *Caspase-8* in hepatic inflammation during EtOH-induced steatohepatitis. It has been previously shown that the Lieber-DeCarli diet induces a limited inflammatory response.^10^ Consistent with the histological findings, both EtOH-fed WT and Casp8^Δhepa^ exhibited a similar moderate increase in hepatic CD45^+^, CD11b^+^ and CD3^+^ expressing cells (Figures 2c–e,Supplementary Figures 3a–c). This was associated with a decrease in mRNA expression of the anti-inflammatory cytokine IL-10 and a slight increase of the pro-inflammatory chemokine CCl2 (Supplementary Figures 3d–e).

Altogether, based on the serum transaminases levels, the histopathological changes and the inflammatory response, our findings suggest that *Caspase-8* deficiency does not prevent alcohol-induced liver injury.

### Deletion of Caspase-8 in hepatocytes leads to attenuated hepatic steatosis after chronic EtOH- challenge

**Figure 3:**
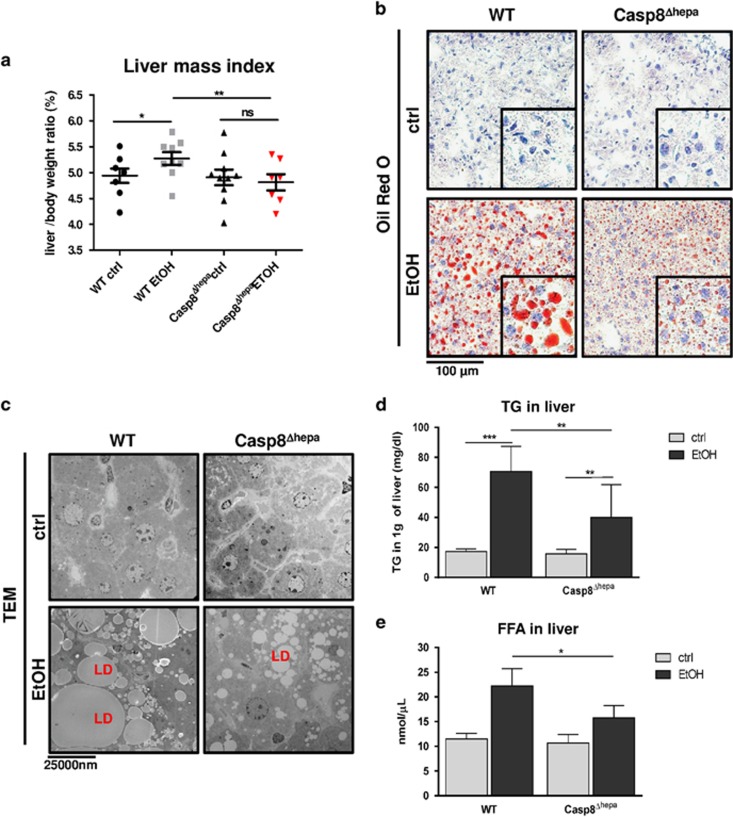
Loss of *Caspase-8* protects hepatocytes from EtOH-induced steatosis. WT (*n*=8–10) and Casp8^Δhepa^ mice (*n*=7) were analyzed after feeding with ctrl or EtOH Lieber-DeCarli diet for 8 weeks. (**a**) Liver weight/body weight ratio. (**b**) Representative Oil Red O staining of liver cryosections from Casp8^Δhepa^ and WT livers. (**c**) Ultrastructural analysis of lipid droplet (LD) accumulation in hepatocytes intake using TEM. (**d**) Hepatic triglyceride (TG) levels in livers of WT and Casp8^Δhepa^ mice. (**e**) Levels of free fatty acids (FFA) in whole liver lysates from WT and Casp8^Δhepa^ mice. *P<0.05, **P<0.01; n.s.: not significant

Steatosis in the liver is a common and important feature of ALD. Therefore, we next investigated whether depletion of *Caspase-8* in hepatocytes may influence the progression of alcohol-induced steatosis. After 8 weeks of EtOH treatment, WT mice exhibited a significantly higher liver/body weight ratio compared to Casp8^Δhepa^ animals (Figure 3a). Explanted livers were subsequently stained with Oil Red O to visualize neutral lipids. In EtOH-treated WT liver sections, numerous lipid-filled vacuoles were present throughout the parenchyma, indicating extensive hepatic macrosteatosis. In contrast, Casp8^Δhepa^ mice revealed a significantly reduced amount of lipid inclusions as demonstrated by morphometric analysis and quantification of Oil Red-O-positive areas in tissue (Figure 3b,Supplementary Figure 4a). Consistently, transmission electron microscopy (TEM) revealed large and aggregated lipid droplets in EtOH-treated WT mice and decreased lipid deposition in Casp8^Δhepa^ animals (Figure 3c).

In line with our results, we observed significantly reduced levels of triglycerides (TG) in the livers of EtOH-fed Casp8^Δhepa^ mice compared to controls (Figure 3d). Simultaneously we observed low levels of hepatic free fatty acids (FFA, Figure 3e). Based on these findings, we propose decreased FFA uptake as a feature of Casp8^Δhepa^ mice which is further supported by significantly reduced expression of translocase CD36 in EtOH-treated Casp8^Δhepa^ livers compared to EtOH-treated WT. Consistently, *PPARγ*, an upstream regulator of CD36, was also significantly less induced by EtOH in Casp8^Δhepa^ animals (Supplementary Figures 4b–c).

Altogether, these results indicate that *Caspase-8* depletion considerably improved EtOH-induced hepatic steatosis via decreased FFA uptake.

### Depletion of Caspase-8 does not prevent EtOH-induced cell death

**Figure 4:**
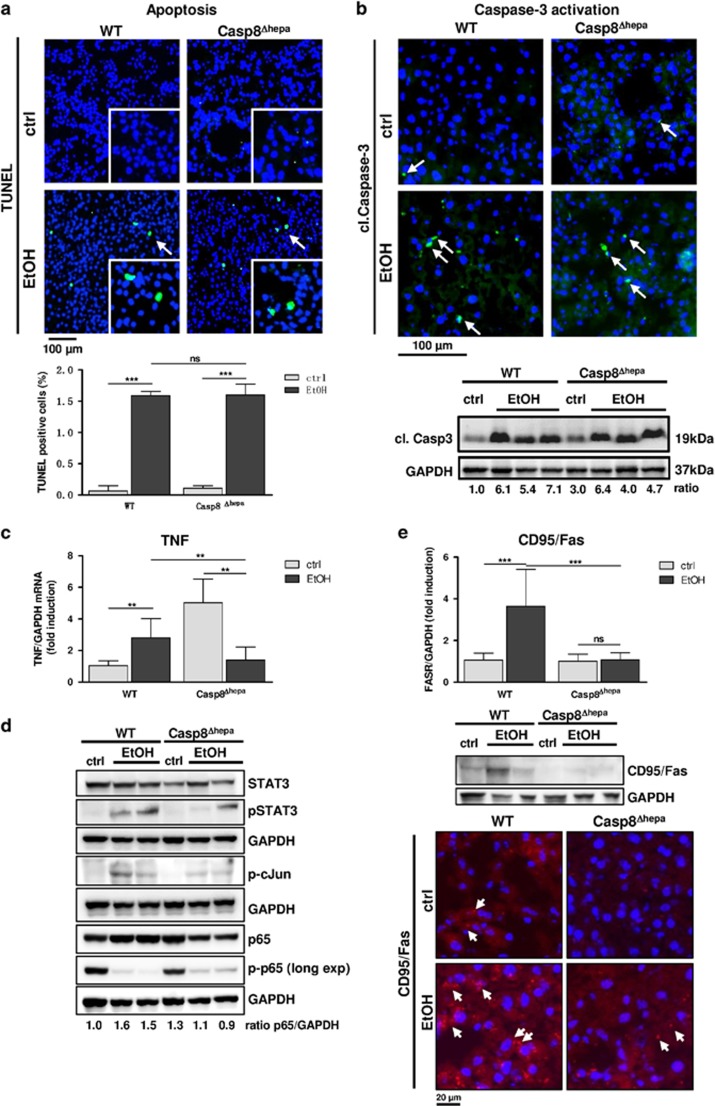
*Caspase-8* is dispensable for EtOH-induced apoptosis. WT and Casp8^Δhepa^ mice were fed with isocaloric (ctrl) or Lieber-DeCarli (EtOH) diet for 8 weeks. (**a**) Analysis of cell death by TUNEL staining (top) and quantification (bottom). Apoptotic cells are stained in green (arrows); total cells were counter-stained with DAPI (blue). (**b**) Analysis of Caspase-3 activation. Top: liver cryosections were stained with an antibody specific for activated (i.e. cleaved; cl.) Caspase-3. Cytoplasmic, activated Caspase-3 is stained in green and highlighted with arrows; total nuclei are counter stained with DAPI (blue). Bottom: immunoblot analysis of cleaved (cl.) Caspase-3 in the liver of WT and Casp8^Δhepa^ mice (bottom). Ratio: normalization of Caspase-3 expression was performed by densitometry. Values are given in arbitrary pixel units and were calculated as fold induction compared to WT (ctrl). (**c**) Determination of TNF gene expression by qPCR. (**d**) Expression and activation analysis of TNF-related immediate downstream factors STAT3, cJun and NF-*κ*B by immunoblot from whole liver lysates. p-p65 membrane was over exposed (Long exp) to detect weak basal expression. Ratio: normalization of p65 expression by densitometry. (**e**) Expression analysis of Fas receptor (CD95/FasR) by qPCR analysis of mRNA expression (top), immunoblot analysis (middle) and immunofluorescence staining (bottom, red, arrows). **P<0.01; ***P<0.01; n.s.: not significant

Next, we examined the contribution of Caspase-8 for EtOH-mediated hepatocellular apoptosis. EtOH feeding induced slight apoptotic cell death in livers of WT – and to the same extent in Casp8^Δhepa^ mice as evidenced by TUNEL-staining (Figure 4a). These results suggest that Caspase-8 is not the main driver of apoptosis in ALD. Death of hepatocytes triggers compensatory proliferation in surrounding cells to maintain tissue homeostasis. The analysis of Ki-67 expression revealed that alcohol feeding triggered mild and similar cell cycle activation in both experimental groups (Supplementary Figures 5a–b). Of note, proliferation was detected mainly in hepatocytes (Supplementary Figure 5c).

To explain the identical rate of apoptosis in WT and Casp8^Δhepa^ livers, we measured Caspase-3 activity, which is one of the common effector caspases in extrinsic and intrinsic apoptosis. In WT mice, Caspase-3 was strongly activated upon EtOH uptake showing focal accumulation in liver tissue (Figure 4b). In Casp8^Δhepa^ mice we revealed increased basal Caspase-3 activity but equally pronounced induction after EtOH feeding as in WT mice (Figure 4b). This finding indicates that loss of Caspase-8 may activate an alternative pro-apoptotic pathway besides the Caspase-8–Caspase-3 axis. We thus investigated pro-apoptotic signals upstream of Caspase-8 such as Fas and TNF.

We could not detect any significant EtOH-related upregulation of TNFR1 expression in both groups of mice (Supplementary Figure 5d). However, basal TNF expression was already significantly elevated in Casp8^Δhepa^ mice, consistently with our previous studies.^9^ EtOH-feeding reverted TNF expression in Casp8^Δhepa^ mice to baseline levels of untreated WT mice (Figure 4c). We thus investigated the activation kinetics of the TNF-downstream factors NF-κB, STAT3 and c-Jun after ethanol challenge by immunoblot analysis (Figure 4d). NF-*κ*B activation (measured by p65 phosphorylation) was basically undetectable in both EtOH-fed WT and Casp8^Δhepa^ mice, correlating with the observed apoptosis induction in both groups. In addition, moderate activation of STAT3 and c-Jun in response to ethanol feeding was observed in WT mice, but only weakly detectable in Casp8^Δhepa^ mice, in agreement with the absence of TNF. Chronic alcohol feeding also induced moderate expression of the CD95/Fas receptor in WT mice, while we did not observe induction of CD95 in EtOH-fed Casp8^Δhepa^ mice, which was confirmed by qPCR, IF staining and immunoblot (Figure 4e). Altogether, major upstream extrinsic death signals are completely switched off in EtOH-fed Casp8^Δhepa^ mice, suggesting that intrinsic signals contribute to apoptotic cell death in this setting.

**Figure 5:**
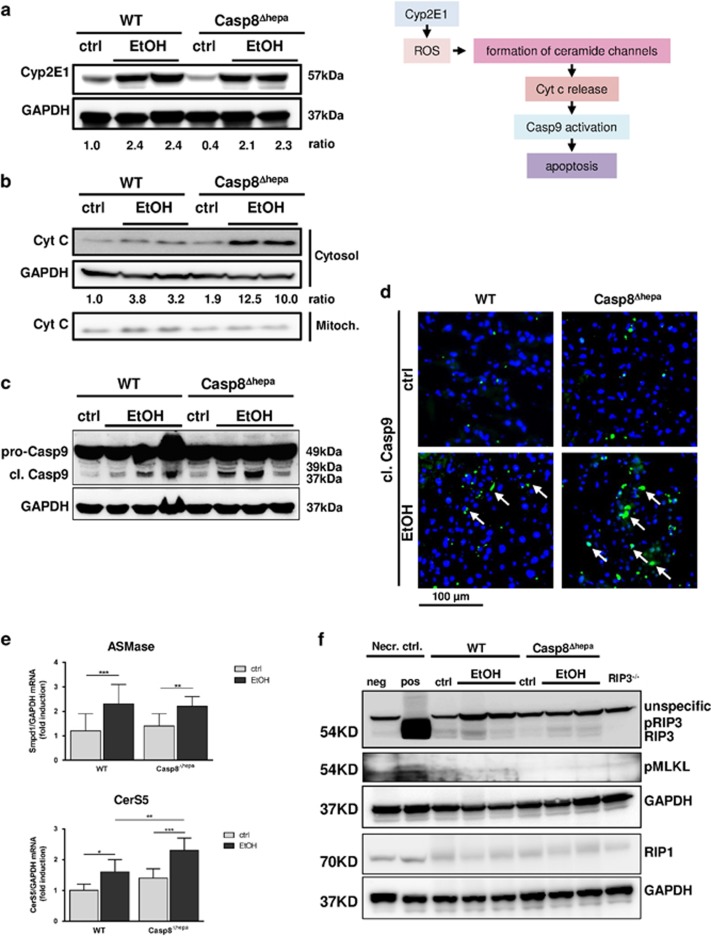
Loss of *Caspase-8* enforces intrinsic apoptosis signaling after EtOH feeding. (**a**) Left: Immunoblot analysis of CYP2E1 in the liver of WT and Casp8^Δhepa^ mice kept on Lieber-DeCarli diet for 8 weeks. Right: Rationale for the subsequent measurements. We hypothesized that ethanol-driven apoptosis in Casp8^Δhepa^ mice could involve de-regulated ceramide biosynthesis and enhanced cytochrome c release from mitochondria thereby inducing increased Caspase-9-mediated intrinsic apoptosis. Accordingly, the indicated intermediate steps were systematically investigated by immunoblot analysis, immunofluorescence stainings and qPCR. (**b**) Western blot analysis of cytochrome c levels in cytosolic and mitochondrial liver fractions. (**c**) Immunoblot analysis of cleaved Caspase-9 in the liver of WT and Casp8^Δhepa^ mice. Quantification of Western blots was performed by densitometry. (**d**) Assessment of Caspase-9 activation *in situ* through fluorescence microscopy. Liver cryosections were stained with an antibody specific for activated (i.e. cleaved; cl.) Caspase-9. Cytoplasmic, activated Caspase-9 is stained in green and highlighted with arrows; total nuclei are counter stained with DAPI (blue). (**e**) qPCR analysis of ASMase and CerS5 mRNA expression. *P<0.05**P<0.01; ***P<0.01. (**f**) Analysis of hepatic necroptosis by immunoblot analysis of RIP3, RIP1 and pMLKL expression. As a positive control for necroptosis (Necr. ctrl., pos), murine embryonic fibroblasts (MEF) were co-stimulated with the pan-caspase inhibitor Z-VAD-FMK (20 *μ*M) and TNF (100 ng/ml) resulting in substantial induction of RIP3 and necroptotic cell death. As a negative control for necroptosis, proteins from untreated MEF (Necr. ctrl., neg) and a whole liver extract from RIP3 knockout mice (RIP3^−/−^) were used

Consequently, we investigated the activation of the intrinsic apoptosis cascade during diet-induced ALD in Casp8^Δhepa^ mice. The mechanism responsible for alcohol-induced intrinsic apoptosis involves CYP2E1 induction, which oxidizes ethanol to reactive metabolites, producing ROS,^3^ resulting in mitochondrial permeability transition, cytochrome c release and apoptosis induction via Caspase-9 (Figure 5a). Of note, WT and Casp8^Δhepa^ mice showed a comparable increase of CYP2E1 expression (Figure 5a) and ROS production (Supplementary Figure 6) upon EtOH treatment pointing to a normal ethanol metabolism in the absence of *Caspase-8.* However, chronic EtOH application led to a marked release of cytochrome c from the mitochondria to the cytosol in Casp8^Δhepa^ livers, which was barely observed in WT mice (Figure 5b). Consistently, this resulted in pronounced activation of the initiator Caspase-9 in livers of EtOH-fed Casp8^Δhepa^ animals (Figures 5c and d).

The mitochondrial permeability tightly correlates with ceramide’s ability to form large channels in the outer phospholipid membrane. We therefore determined the expression levels of main mitochondrial enzymes involved in ceramide synthesis and found increase of ASMase and CerS5 in the livers of EtOH-treated WT and Casp8^Δhepa^ mice (Figure 5e). Moreover, EtOH feeding induced significantly stronger upregulation of CerS5 in livers of EtOH-fed Casp8^Δhepa^ animals, hinting at intensified mitochondrial permeability transition.

We also analyzed the mitochondrial morphology in livers of WT and Casp8^Δhepa^ mice following EtOH-treatment by TEM. In WT mice, a high number of small, electron-dense, compact mitochondria with organized double membranes and cristae were easily recognizable in hepatocytes. After 8 weeks of EtOH-diet, mitochondria exhibited marked round shape, swelling, disruption of double layer structure and disorganized cristae in both WT and in Casp8^Δhepa^ hepatocytes (Supplementary Figure 7).

The main mechanisms of cell death during ALD progression have been related to apoptosis, classical necrosis or necroptosis.^11, 12^ To test whether ablation of Caspase-8 may predominantly trigger necroptotic cell death upon EtOH challenge, as recently suggested,^12^ we assessed the expression of the pro-necroptotic Receptor-interacting serine/threonine-protein kinase-1 and -3 (RIP1, RIP3) and the phosphorylated pseudokinase Mixed lineage kinase domain-like protein (MLKL). As a positive control for necroptosis, we used murine embryonic fibroblasts (MEF) co-treated with the pan-caspase inhibitor Z-VAD-FMK and TNF resulting in substantial necroptotic cell death. As a negative control for necroptosis, a whole liver extract from RIP3 knockout mice (RIP3^−/−^) was used.

EtOH feeding resulted in a slight induction of RIP3 expression in WT mice and to even a lesser extent in Casp8^Δhepa^ animals, while RIP3 was massively upregulated in necroptotic MEFs in agreement with earlier reports (Figure 5f). Noticeably, we found slight basal expression of RIP1 in all samples without further induction in either experimental group. However, execution of necroptosis essentially depends on phosphorylation of the RIP3 downstream target MLKL. Importantly, we could not detect enhanced MLKL phosphorylation after EtOH feeding exceeding background levels in either group, suggesting that canonical necroptosis is not involved in alcoholic liver injury, despite moderate RIP3 expression.

Altogether, our data suggest that Caspase-8 depletion in hepatocytes does not prevent apoptosis in ALD, but shifts the pro-apoptotic signaling from an extrinsic pathway towards stronger release of cytochrome c and intrinsic apoptotic cell death signaling.

### Pan-caspase inhibitors rescue hepatocytes from alcohol-induced cell death both *in vitro* and *in vivo*

**Figure 6:**
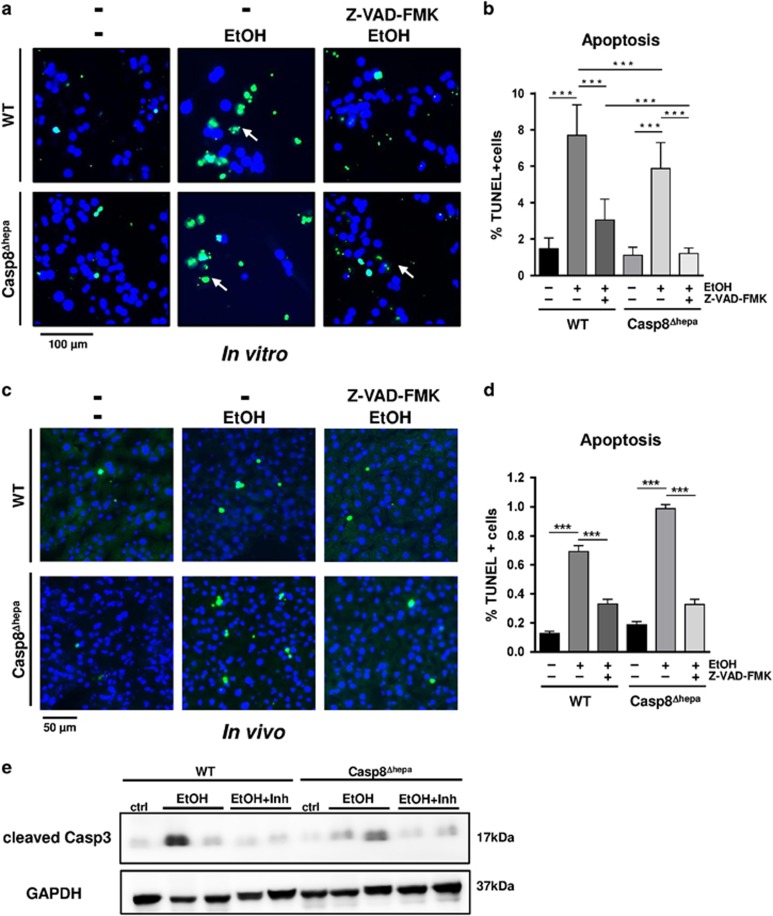
Pan-caspase inhibitors prevent alcohol-induced hepatic apoptosis *in vitro and in vivo*. (**a–b**) I*n vitro* analysis. Primary hepatocytes from WT and Casp8^Δhepa^ mice were isolated, plated and stimulated with 100 mM EtOH for 24 h with or without pan-caspase inhibitor Z-VAD-FMK (**a**) TUNEL staining. Apoptotic nuclei are stained in green. Total nuclei are counter-stained with DAPI (blue). (**b**) Quantification of cellular apoptosis. Data were calculated as percentage of TUNEL-positive cells per magnification field. (**c–e**) *In vivo* analysis. WT and Casp8^Δhepa^ mice (*n*=4–6) were fasted for 6 h and then injected (i.v.) with 20 *μ*g/g body weight of Z-VAD-FMK/8% or solvent control. Subsequently, they were fed with 30% (w/v) EtOH by three equally divided oral gavages in 20-min intervals. All animals were killed 12 h after last EtOH feeding. (**c**) TUNEL staining of liver cryosections. (**d**) Quantification of apoptosis. For each animal, 10 independent magnification fields (× 200) were counted. (**e**) Immunoblot analysis of Caspase-3 activation

Next, we tested the consequences of broad caspase inhibition on EtOH-induced apoptosis *in vitro* using the pan-caspase inhibitor Z-VAD-FMK in primary isolated hepatocytes. EtOH treatment strongly induced hepatocyte apoptosis in WT cells. Hence, *Caspase-8* deletion had only a minor effect on cell viability *in vitro* (Figures 6a and b). Importantly, alcohol-induced hepatic apoptosis in WT and KO hepatocytes was greatly reduced after Z-VAD-FMK treatment.

To validate these *in vitro* findings, we studied Z-VAD-FMK pre-treatment with acute administration of EtOH in *vivo*. First of all, oral gavage of high dose EtOH (6 g/kg body weight) alone resulted in similar apoptosis induction and Caspase-3 activation in both WT and Casp8^Δhepa^ mice (Figures 6c–e) thereby confirming our data from chronic EtOH feeding experiments. Remarkably, pre-administration of the pan-caspase inhibitor provided significant protection in both groups of animals against EtOH-induced liver apoptosis concurring with the *in vitro* data (Figures 6c–e).

Altogether these data suggest that caspase-driven apoptosis in alcoholic liver injury is independent of *Caspase-8* but can be prevented with the administration of less specific pan-caspase inhibitors.

## Discussion

Apoptosis represents the execution of a highly regulated programmed form of cell death initiated by specific stimuli. Around 15 years ago, different studies highlighted the relevance of apoptosis for ALD.^2, 13^ For instance, hepatocyte apoptosis has been robustly reported in different experimental models of EtOH-induced liver injury^14, 15^ and in alcoholic patients in direct correlation with disease severity.^16, 17^

Further investigations suggested that extrinsic, DR-mediated apoptosis is a central event in the pathogenesis of ALD. Indeed, overexpression of CD95/Fas receptor in hepatocytes and its associated Fas ligand (FasL) as well as increased TNF serum levels have been observed in patients with alcohol-induced hepatitis.^18, 19^ Furthermore, chronic EtOH exposure upregulates TNF receptor expression potentially sensitizing hepatocytes to TNF-driven cell death.^20^

However, apoptosis is a complex process and up to now the precise contribution of main key events involved in the apoptotic cascade during ALD progression remains still unclear. Therefore the ultimate aim of the present study was to investigate the role of *Caspase-8* – the most apical caspase implicated in the initiation of the DR-mediated apoptosis – in a well-established experimental model of ALD.

Our initial data indicated a strong presence of *Caspase-8* activation in human and murine ALD supporting the rationale of our study. However, the analysis of mice with hepatocyte-specific genetic depletion of *Caspase-8* challenged with Lieber-DeCarli revealed completely unexpected results, pointing to the need of re-defining the caspase-dependent mechanisms during alcohol-induced liver injury.

First, ablation of *Caspase-8* resulted in reduced alcohol-mediated liver steatosis. Second, the loss of *Caspase-8* did not result in a reduction of overall liver injury or apoptosis. Instead, we found strong evidence that inactivating Caspase-8 shifted a dominant extrinsic death pathway towards strong intrinsic, *Caspase-8* independent apoptosis.

Our finding that loss of Caspase-8 prevents EtOH-induced hepatosteatosis is surprising at a first glance. However, it supports our previous study showing reduced steatosis, hepatic lipid storage and accumulation of FFAs in Casp8^Δhepa^ mice fed with a steatohepatitis-inducing methionine-choline-deficient diet.^21^ We then reasoned that Caspase-8 is not only involved in apoptosis signaling, but also in the regulation of hepatic fat metabolism. This concept is supported by previous transcriptome analysis that demonstrated differential regulation of metabolic genes in Casp8^Δhepa^ mice.^21^ It is tempting to speculate that the protease activity of Caspase-8 is mechanistically required for the post-translational modification of factors involved in the transcriptional regulation of metabolic enzymes. However, the identification of such factors was beyond the scope of the present study and warrants further investigation. Although Caspase-8 inactivation in hepatocytes did not exert a hepatoprotective effect after 8 weeks of alcohol feeding, we cannot exclude the possibility that reduced steatosis might be beneficial within a longer observational period as a result of decreased hepatic ROS production. Yet this would be an indirect rather than a direct effect of Caspase-8 inhibition.

Extensive evidence from the past years has demonstrated that inactivation of *Caspase-8* may sensitize cells or organs to an alternative form of programmed cell death termed necroptosis.^22^ The molecular requirements and definitions of necroptosis have been recently proposed by the Nomenclature Committee on Cell Death.^23^ Accordingly, necroptosis requires at least DR signaling, inhibition of *Caspase-8*, and activation of the receptor-interacting kinases RIP1 and/or RIP3. In addition, it has been shown that phosphorylation of MLKL by RIP3 is critical for necroptotic cell death.^24, 25^ The group of Laura E. Nagy^12^ recently showed that RIP3 is induced by EtOH uptake, while mice lacking RIP3 were protected from ethanol-induced steatosis and hepatocyte injury. Interestingly, RIP3 knockout mice still undergo apoptosis. Concomitant with this study, we detected slight RIP3 induction upon alcohol feeding in WT and to a lesser extent in Casp8^Δhepa^ mice. However, we did not detect signs of necroptosis (i.e. increased liver injury, necrotic tissue morphology, MLKL phosphorylation) in either group compared to established positive controls. We thus conclude that ablation of *Caspase-8* does not induce RIP-dependent necroptosis in our murine model of ALD.

We and others have investigated the consequences of Caspase-8 depletion on necroptosis induction in a variety of disease models revealing divergent effects. In models of Fas- or LPS/GalN-induced apoptosis, simple steatosis or after partial hepatectomy, deletion of Caspase-8 turned out to be hepatoprotective,^9, 21, 26^ while in strong inflammatory injury models such as Concanavalin A treatment, NEMO/IKKγ deletion or MCD diet, Caspase-8 deficiency was associated with induction of necroptosis.^9, 27^ In our present study, we have demonstrated that feeding a Lieber-DeCarli diet is not related to a major inflammatory response. We conclude that these divergent findings are best explained by the hypothesis that absence of Caspase-8 predominantly predisposes to necroptosis in a strong inflammatory liver environment and thus not in the Lieber-DeCarli model. In addition, our present data revealed that TNF and Fas signaling pathways were largely switched off in Casp8^Δhepa^ mice following alcohol feeding, which was confirmed by several independent methods. Although the underlying mechanism for the positive EtOH/Caspase-8/TNF/Fas feedback loop remains currently unclear, it can be assumed that lack of DR signaling contributes to the absence of necroptosis in EtOH-fed Casp8^Δhepa^ mice.

**Figure 7:**
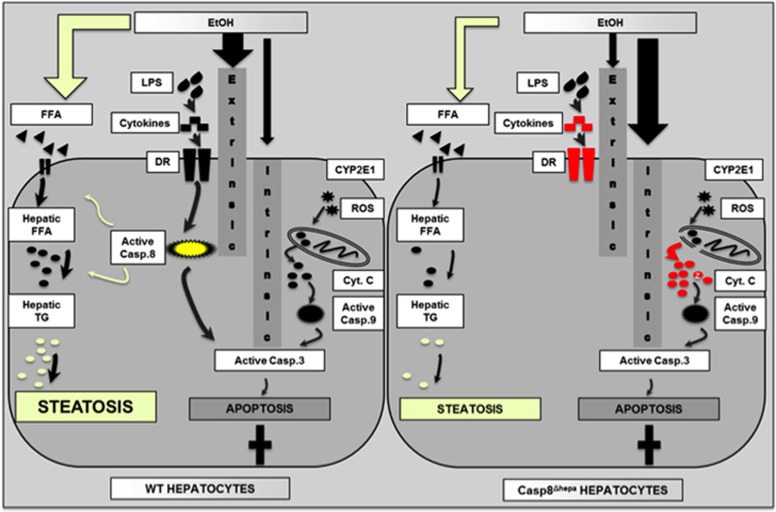
Simplified graphical scheme representing the effect of *Caspase-8* depletion on hepatic apoptosis and steatosis in the pathogenesis of ALD. Left: In WT hepatocytes, EtOH exposure induces both extrinsic and intrinsic apoptosis with a favorable shift towards Caspase-8-dependent extrinsic signaling. In addition, Caspase-8 plays an unexpected and presumably non-apoptotic role in the metabolism of hepatic free fatty acids (FFA) and triglycerides (TG) thereby contributing to pronounced liver steatosis. Right: Inhibition of *Caspase-8* results in abrogation of the extrinsic apoptosis pathway. Instead, alcohol-dependent reactive oxygen species (ROS) result in enhanced mitochondrial permeability transition, enhanced cytoplasmic cytochrome c release and enhanced intrinsic apoptosis signaling eventually leading to equal net apoptosis. However, lack of Caspase-8 is associated with reduced production of FFA and TG eventually leading to attenuated liver steatosis

Altogether, our study provides new insights into the pro-apoptotic signaling pathways during ALD initiation and progression (Figure 7). We suggest that alcohol represents a ‘mixed’ type of apoptotic agent and may employ both pathways of apoptosis signalling (i.e. intrinsic and extrinsic). We propose that in ALD patients and WT mice, *Caspase-8* in hepatocytes is dominantly involved in the pro-apoptotic response towards alcohol and directly activates effector caspases such as caspase-3. Accordingly, activation of the intrinsic initiator caspase, caspase-9, is moderate. In turn, simultaneous inactivation of *Caspase-8* and chronic alcohol intake results in a switch from extrinsic type I apoptosis towards intrinsic, mitochondria-dependent cell death as evidenced by enhanced cytochrome c release from Casp8^Δhepa^ mitochondria, enhanced upregulation of Ceramide Synthase 5 (CerS5) and pronounced proteolytic activation of Caspase-9. CerS5 is an important pro-apoptotic ceramide, whose expression is upregulated during ROS and DNA damage, and is an essential trigger for mitochondria-mediated apoptosis.^28, 29, 30^ As it has been recently shown, ceramide form large, stable channels in the mitochondrial outer membrane, accelerate permeability pathways and thereby facilitate intrinsic apoptosis. It is therefore tempting to assume that the switch from extrinsic to intrinsic alcoholic apoptosis in absence of Caspase-8 may be in part due to increased levels of pro-apoptotic ceramides as illustrated in Figure 7.

Our findings support the concept that only simultaneous blockage of both intrinsic and extrinsic apoptosis pathways might be beneficial against EtOH-induced hepatic cell death. Consistently, we found that administration of a pan-caspase inhibitor substantially abolished apoptosis in EtOH-treated primary hepatocytes *in vitro* and in an alcohol-binge-drinking model *in vivo* in a Caspase-8 independent manner. These findings are in agreement with earlier reports,^6, 31^ demonstrating that this therapeutic strategy has been already found to be beneficial for the treatment of other chronic liver diseases such as non-alcoholic steatohepatitis^32^ or HCV.^2^

In summary, we provide evidence that the extrinsic, Caspase-8-dependent apoptosis pathway is dispensable for the initiation and progression of ALD as hepatocytes are capable to switch to intrinsic cell death upon *Caspase-8* deficiency. Therefore, only a combined approach using a pan-caspase inhibitor completely blocking caspase activity in hepatocytes might be an optimal approach for the treatment of ALD.

## Material and methods

### Human liver samples

Human liver explants from 10 ALD patients with alcoholic end-stage liver cirrhosis were investigated (Supplementary Table 1). Control biopsies were taken from three healthy subjects with histologically normal livers, who underwent liver biopsy because of mildly elevated liver enzymes. The study protocol was approved by the Ethics Committee of University Hospital RWTH Aachen, and conducted according to the principles expressed in the Declaration of Helsinki.

### Maintenance of mice and animal experimentation

All animal experiments in this project were designed and performed in accordance with the German and Spanish laws and regulations on animal protection. All procedures were approved by the authority for nature, environment and consumer protection of the state North Rhine-Westphalia (LANUV, Germany) and by the Consejería de Medio Ambiente, Administración Local y Ordenación del Territorio de la Comunidad de Madrid (Madrid, Spain). Mice were kept in a specific pathogen-free facility in a temperature-controlled room with 12-h light/dark cycles and free access to food and water. For the experiments, we exclusively used animals of male gender bred in a C57BL/6 background. The generation of hepatocyte-specific Caspase-8 knockout mice (Casp8^Δhepa^) using the cre/loxP system has been described recently.^9^ For all experiments, cre-negative Casp8^f/f^ littermates were used as controls.

RNA isolation and quantitative Real-Time PCR Analysis (qPCR). *qPCR* was performed as described recently.^33^ Samples were collected randomly. All measurements were normalized using GAPDH expression as an internal standard and calculated as fold induction in comparison to untreated controls. The primer sequences are shown in Supplementary Table 2.

### Immunohistochemistry and immunofluorescence stainings of liver sections

Hepatic tissue was fixed in Methacarn immediately after extraction, embedded in paraffin, sectioned and subjected to staining for H&E and Oil Red O as explained before.^21, 33, 34^ Details on the methodology of IHC and IF can be found in previous publications from our laboratory. The source of the commercially available antibodies used for the immunohistochemistry is listed in Supplementary Table 3.

All samples were collected randomly, blindly analyzed and documented using an Imager Z1 microscope together with Axiovision software (Carl Zeiss, Jena, Germany). Ki-67 and CD-11b stainings were quantified by determination of the number of positive cells/positive nuclei in at least 10 randomly selected magnification fields at × 100 magnification. Quantitative determination of liver steatosis in mice was assessed histologically by quantification of Oil Red-O-positive areas using free NIH ImageJ software (http://imagej.nih.gov/ij/, National Institutes of Health, Bethesda, MD, USA) as previously described.^35^

### Immunoblot analysis

Immunoblot analysis was carried out according to standard procedures.^33^ Samples were collected randomly. Membranes (Whatman Protran) were probed with antibodies listed in Supplementary Table 4. As secondary antibodies, anti-rabbit-HRP and anti-mouse-HRP (Santa Cruz, Santa Cruz, CA, USA) were used. For all western blots, GAPDH probing was performed as internal control using an antibody from AbD seroTec (Kidlington, UK).

### Transmission electron microscopy

TEM was carried out using Zeiss Leo 906 electron microscope as described before.^36^

#### General methodology

ALT, AST and serum TG were processed by the Central Laboratory Facility at University Hospital RWTH Aachen. FFA was measured in liver tissue using a ready-to-use kit (Abcam, UK, Free Fatty Acid Quantification Kit, ab65341) according to the provider’s instruction.

### Mitochondria isolation

For mitochondria isolation exclusively freshly harvested livers were used. Mice used for the experiment were fasted overnight. Mice were killed and the liver was explanted, immersed in 50 ml of ice-cold IBc buffer and rinsed 4–5 times or until the blood was removed. The liver was minced into small pieces with a scalpel and further homogenized on ice in 5 ml of fresh IBc buffer. The homogenate was transferred into a 50 ml tube and centrifuged for 10 min at 600 × *g* and 4 °C. The remaining supernatant was transferred into a glass centrifuge tube and centrifuged for 10 min at 7000 × *g* and 4 °C. The subsequent supernatant contained the cytosolic fraction and was stored at -80 °C. The pellet containing the mitochondria was washed with 3 ml of IBc buffer and centrifuged again for 10 min at 7000 × *g* and 4 °C. The supernatant was then discarded; the pellet containing mitochondria was re-suspended in NP40 lysis buffer and stored at −80 °C.^37^

### Application of pan-caspase inhibitor *in vitro* and *in vivo*

The pan-caspase inhibitor Z-VAD-FMK was obtained from APExBio (Houston, TX, USA). For *in vitro* application, primary hepatocytes from WT and Casp8^Δhepa^ mice were isolated, plated on Petri dishes and stimulated with 100 mM EtOH for 24 h with or without 20 *μ*M pan-caspase inhibitor Z-VAD-FMK. For *in vivo* treatment, WT and Casp8^Δhepa mice^ (*n*=4–6) were fasted for 6 h and then injected (i.v.) with 20 *μ*g/g body weight of Z-VAD-FMK/8% DMSO or solvent alone. Twenty minutes after injection, animals were fed with 30% (w/v) ethanol at a total accumulative dosage of 6 g/kg body weight by three equally divided oral gavages in 20-min intervals. All animals were killed 12 h after last EtOH feeding and analyzed for markers of apoptosis.

### Statistical analysis

Data are expressed as mean±standard deviation of the mean. Statistical significance was determined by two-way analysis of variance followed by a Student’s *t*-test. *P*-values for significance are indicated as follows: *P<0.05; **P<0.01; ***P<0.001.

## Publisher’s Note

Springer Nature remains neutral with regard to jurisdictional claims in published maps and institutional affiliations.
