# Supplementary figures and images for: Inhibition of Caspase-8 does not protect from alcohol-induced liver apoptosis but alleviates alcoholic hepatic steatosis in mice
Source: Cell Death Dis. 2017 Oct 26;8(10):e3152–. doi: 10.1038/cddis.2017.532 (PMC5680911; doi:10.1038/cddis.2017.532)

**a****ALD****cl. Casp8****Isotype ctrl**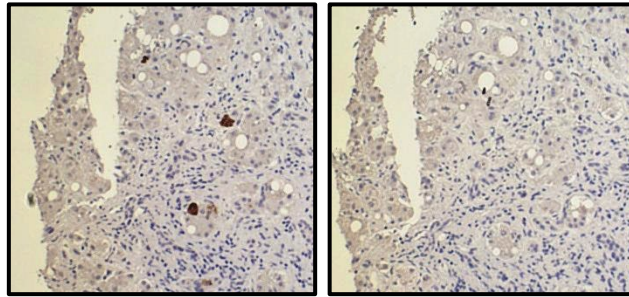

50 μm

**b****EtOH****WT****Casp8<sup>Δhepa</sup>****cl. Casp8**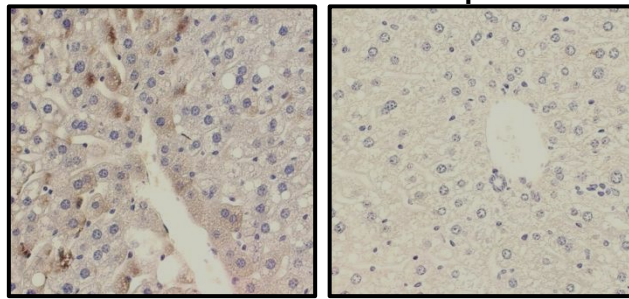

50 μm

**a**

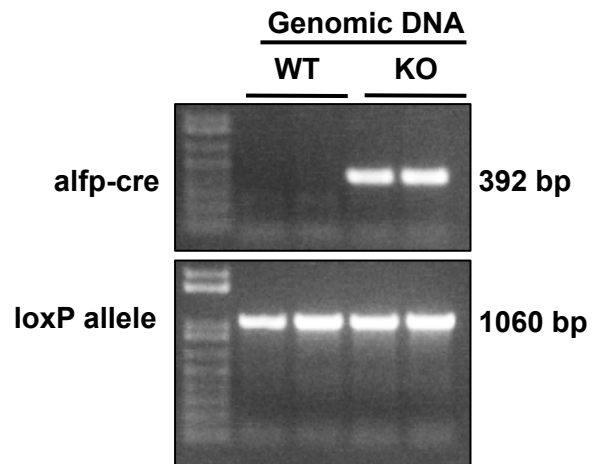

**b**

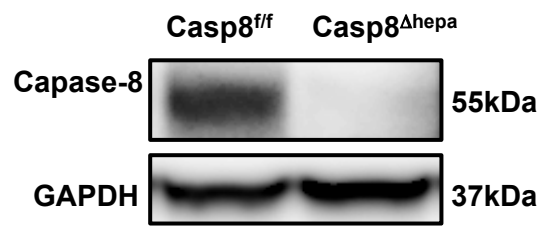

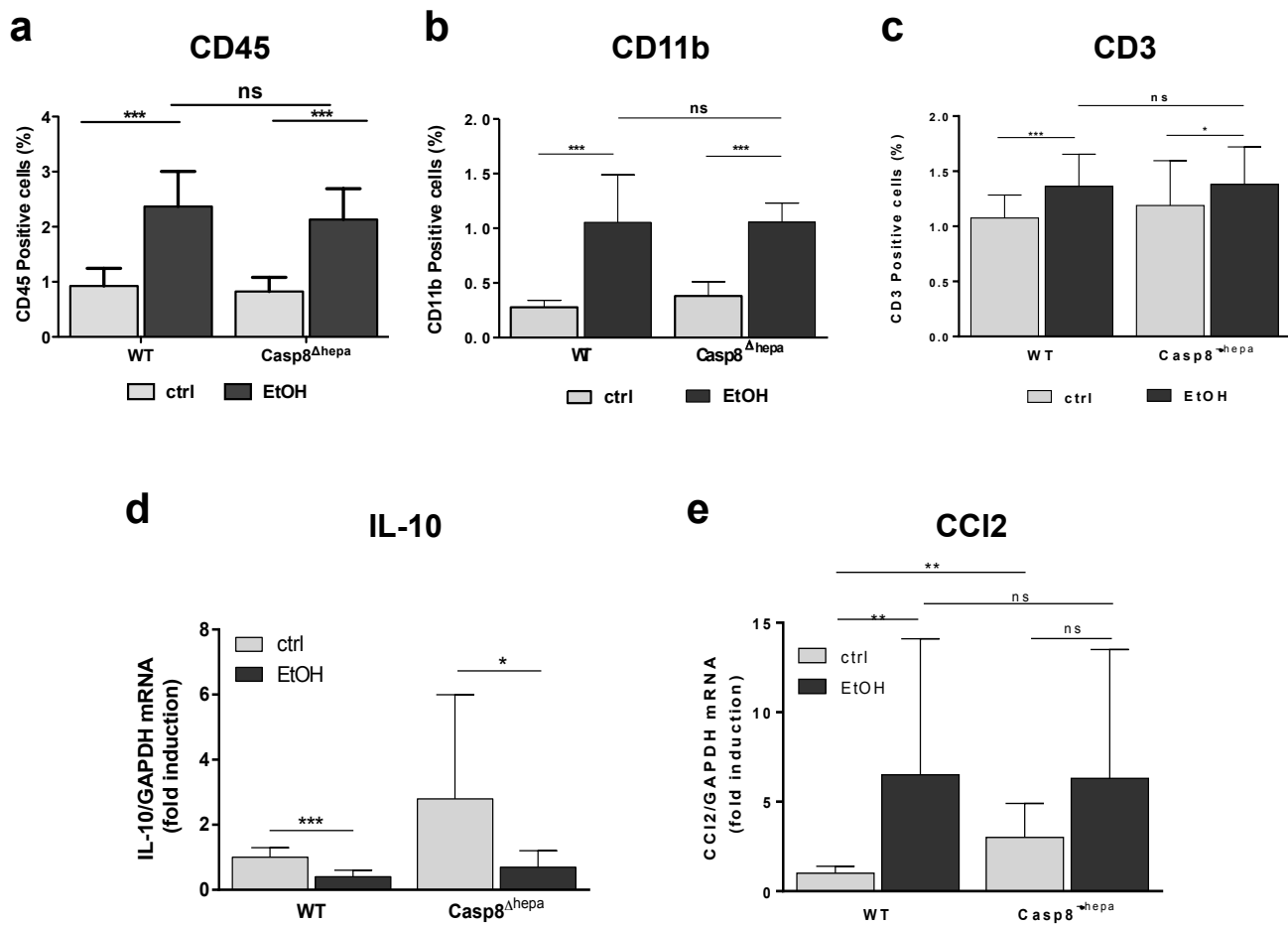

**a****Steatosis**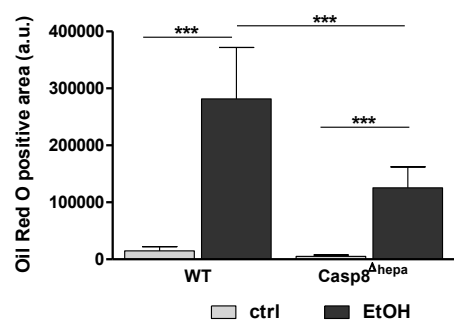**b****CD36**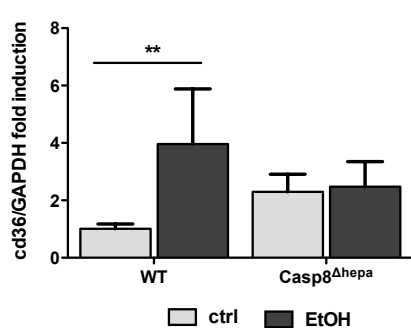**c****PPAR $\gamma$** 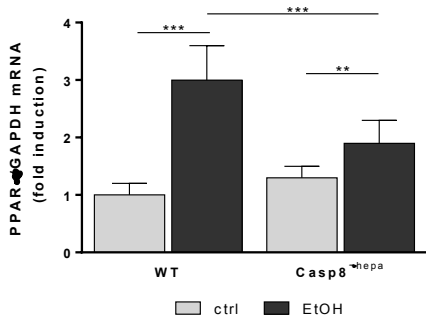

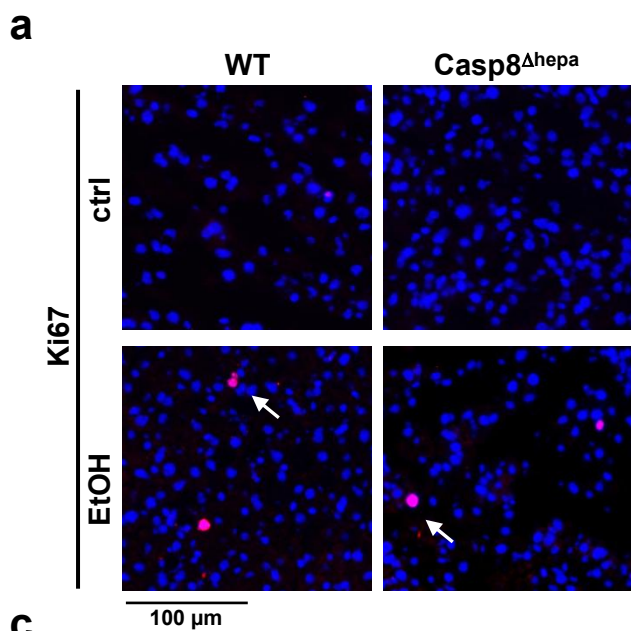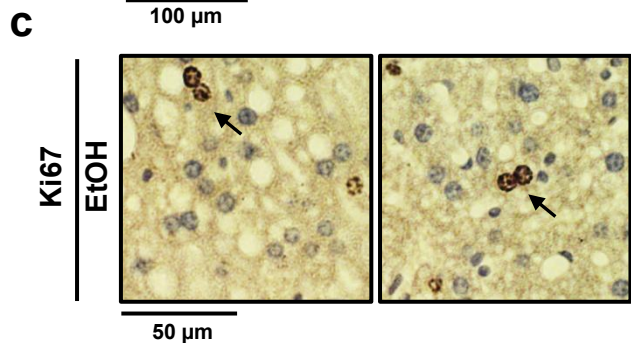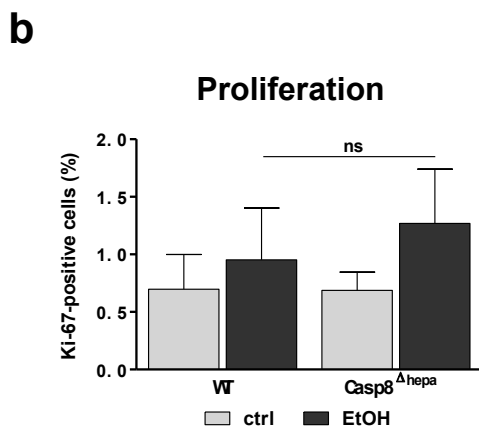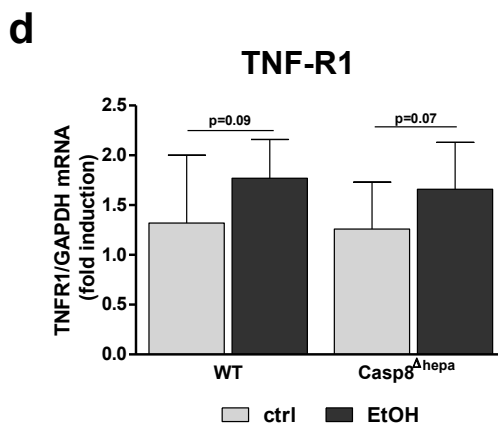

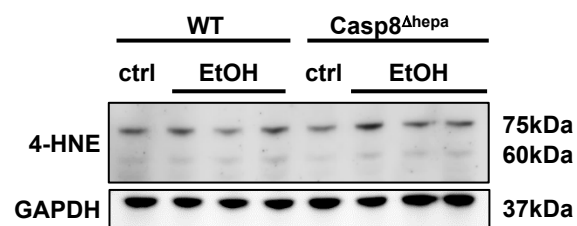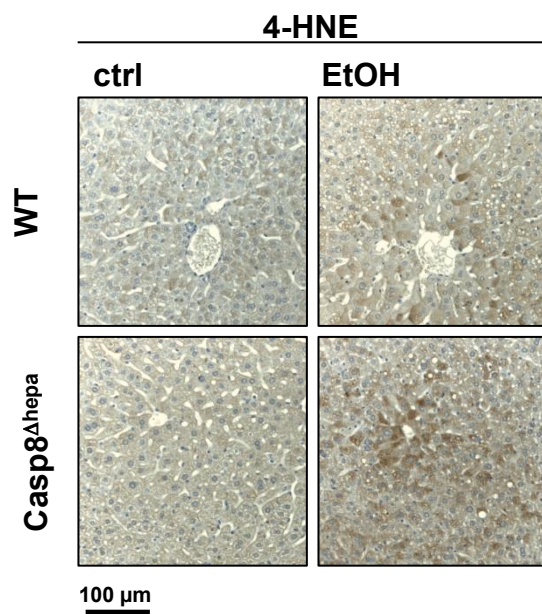

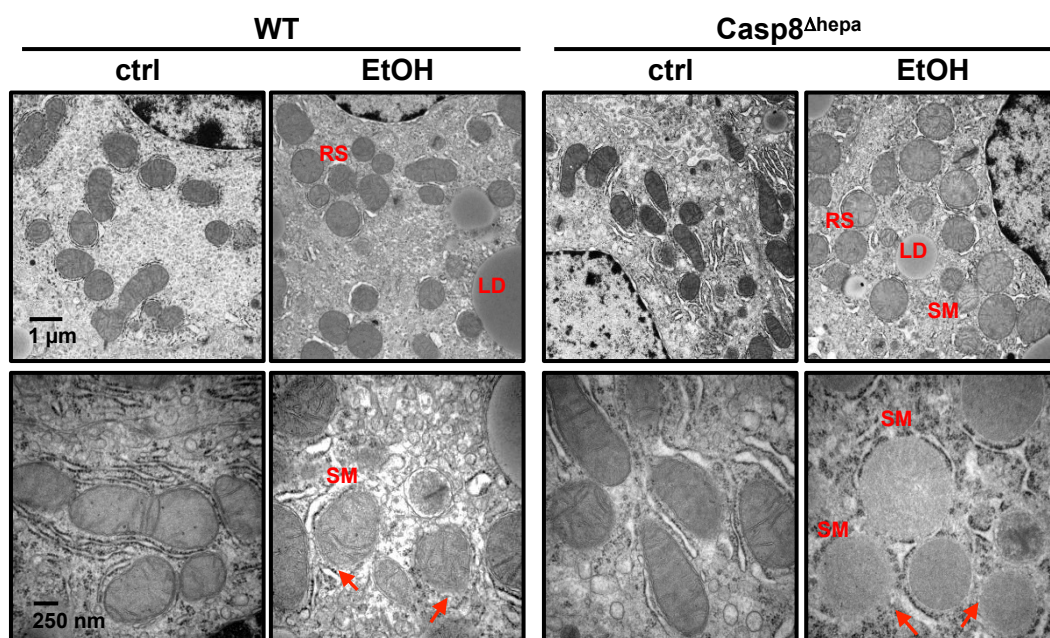

Supplement: Supplementary Figures [file cddis2017532x1.pdf]
