# Supplementary material for: Inhibition of Caspase-8 does not protect from alcohol-induced liver apoptosis but alleviates alcoholic hepatic steatosis in mice
Source: Cell Death Dis. 2017 Oct 26;8(10):e3152–. doi: 10.1038/cddis.2017.532 (PMC5680911; doi:10.1038/cddis.2017.532)
Supplement: Supplementary Figure Legends and Tables [file cddis2017532x2.docx]

**Supplementary materials to:**

**Inhibition of Caspase-8 does not protect from alcohol-induced liver apoptosis but alleviates alcoholic hepatic steatosis in mice**

**Supplementary Figure Legends**

Supplementary Figure 1. **Activation of Caspase-8 in human and murine ALD.** (**a**) Liver sections from ALD patients were incubated with an antibody specific for activated Caspase-8 (cl. Casp8) or rat IgG as isotype control. (**b**) Immunostaining specific for cleaved (i.e. activated) Caspase-8 (brown) of paraffin sections of WT mice treated with Lieber-DeCarli diet for 8 weeks.

Supplementary Figure 2. **Efficiency of Caspase-8 deletion in hepatocytes.** (**a**) Representative genotyping of mice with a floxed Caspase-8 allele and an alfp-cre transgene (KO) and cre-negative controls (WT) was performed from genomic tail DNA. (**b**) Western blot analysis of Caspase-8 expression in primary hepatocytes isolated from WT and Casp8^Δhepa^ mice.

Supplementary Figure 3. **Deletion of Caspase-8 does not affect hepatic inflammation after chronic alcohol uptake. (a-c)** Quantifications of immunofluorescence stainings for **(a)** CD45 (leukocyte-specific), **(b)** CD11b (myeloid cells) and **(c)** CD3 (T-cells) shown in Figure 2c-e. The number of positive-stained cells was quantified and calculated as percentage of total cells counterstained with DAPI. (**d-e**) Hepatic expression of the anti-inflammatory marker IL-10 (d) and the pro-inflammatory factor CCl2 (e) was determined by qPCR. GAPDH served as an endogenous control. *: p<0.05; **: p<0.01; ***: p<0.001; ns: not significant.

Supplementary Figure 4. Inhibition of Caspase-8 prevents steatosis after continuous alcohol uptake. (**a**) Quantification of Oil Red O-stained liver tissue area using ImageJ software. (**b-c**) Hepatic mRNA expression of (**d**) CD36, (**e**) PPARγ. *: p<0.05;**: p<0.01; ***: p<0.001.

Supplementary Figure 5. EtOH treatment induces **slight compensatory proliferation independently of Caspase-8.** (**a**) Immunofluorescence staining of Ki-67 on frozen liver sections. Nuclear Ki-67 expression (red, arrows) indicates overall cell proliferation. (**b**) Proliferation was quantified as percentage Ki-67-positive nuclei per high magnification field. (**c**) Ki-67 immunostaining of liver paraffin sections. Nuclear Ki-67 expression (brown nuclei, arrows) indicates overall compensatory cell proliferation of hepatocytes after EtOH intake. (**d**) qPCR analysis of TNFR1 mRNA expression; ns: not significant.

**Supplementary Figure 6. Inhibition of Caspase-8 is not associated with marked increase of ROS production after EtOH treatment.** WT and Casp8^∆hepa^ mice were fed with isocaloric (ctrl) or Lieber-DeCarli (EtOH) diet for 8 weeks. Upper panel: Immunoblot showing expression of 4 Hydroxynonenal (4-HNE) serving as a marker for lipid peroxidation and oxidative stress. Please note that only the 75 kDa and 60 kDa aldehydic products are shown. Anti-GAPDH was used for normalizing. Bottom panel: Immunhistochemistry for 4-HNE (brown) on paraffin sections

Supplementary Figure 7. Analysis of mitochondrial morphology in livers of WT and Casp8^Δhepa^ mice following EtOH-treatment by transmission electron microscopy (TEM)**.** Representative TEM images of hepatic mitochondria are shown. Arrows: mitochondrial double membrane disruption; LD: lipid droplets; SM: swollen mitochondria; RS: round shaped mitochondria.

**Supplementary Table 1**: Etiology and fibrosis score of human liver samples used for Caspase-8 expression analysis

| **Sample** | **Etiology** | **Clinical presentation** | **Fibrosis**  **stage** | **Inflammation score** |
| --- | --- | --- | --- | --- |
| **G2** | n.a. | healthy | 0 | 0 |
| **G3** | n.a. | healthy | 0 | 0 |
| **G4** | n.a. | healthy | 0 | 0 |
| **B22** | ALD | decompensated cirrhosis | 4 | 2-3 |
| **B28** | ALD | decompensated cirrhosis | 4 | 2-3 |
| **B29** | ALD | decompensated cirrhosis | 3 | 2 |
| **B30** | ALD | decompensated cirrhosis | 4 | 2-3 |
| **B35** | ALD | decompensated cirrhosis | 4 | 1-2 |
| **B36** | ALD | decompensated cirrhosis | 4 | 2 |
| **B37** | ALD | decompensated cirrhosis | 4 | 2 |
| **B38** | ALD | decompensated cirrhosis | 4 | 1-2 |
| **B39** | ALD | decompensated cirrhosis | 4 | 2-3 |
| **B41** | ALD | decompensated cirrhosis | 4 | 1-2 |

ALD: Alcoholic liver disease; n.a.: not applicable (healthy tissue)

| **Stage** | **Fibrosis score** |
| --- | --- |
| 0 | None |
| 1 | Enlarged fibrotic portal tracts |
| 2 | Periportal or portal-portal septa, but intact architecture |
| 3 | Fibrosis with architectural distortion, no obvious cirrhosis |
| 4 | Probable or definite cirrhosis |

| **Stage** | **Inflammation score** |
| --- | --- |
| 0 | no / minimal inflammation |
| 1 | portal inflammation or lobular inflammation without necrosis |
| 2 | mild periportal inflammation and piecemeal necrosis or focal hepatocellular necrosis |
| 3 | moderate periportal inflammation and piecemeal necrosis or severe focal cell damage |
| 4 | severe periportal inflammation and piecemeal necrosis or bridging necrosis |

**Supplementary Table 2.** Primer sequences used for quantitative real time PCR

| **#** | **Forward** | | **Reverse** |
| --- | --- | --- | --- |
| FasL | TCATTGCACTGGAGGTATGC | | GTTTTCTGAGCCGACCTTTG |
| FasR | GGCTGTGAACACTGTGTTCGC | | GGATGGTCAACAACCATAGGCG |
| GAPDH | TGTTGAAGTCACAGGAGACAACCT | | AACCTGCCAAGTATGATGACATCA |
| IL6 | GCTACCAAACTGGATATAATCAGGA | | CCAGGTAGCTATGGTACTCCAGAA |
| PPARγ | CACAATGCCATCAGGTTTGG | | GCTGGTCGATATCACTGGAGATC |
| TNF | | CCTCTTCTCATTCCTGCTTGTGG | GAGAAGATGATCTGAGTGTGAGG |
| TNFR | | GGAAAGTATGTCCATTCTAAGAACAA | AGTCACTCACCAAGTAGGTTCCTT |
| CerS5 | | gctggcagtgtgcatcttc | tttaatgccaacacggagtg |
| ASMase | | cccgcctgcaaagtcttat | ccacattgggctccttctt |
| CCl2 | | GTGTTGGCTCAGCCGATGC | GACACCTGCTGCTGGTGATCC |
| IL-10 | | GGCTGAGGCGCTGTCATCG | TCATTCATGGCCTTGTAGACACC |

**Supplementary Table 3.** List of commercially available antibody used for IHC and IF

| **Product** | **Manufacturer** | **Catalog No.** | **Dilution** |
| --- | --- | --- | --- |
| CD11b | BD Pharmingen | 550282 | 1：100(IF) |
| Cleaved Casp3 | Cell Signaling Technology | 9661 | 1:500(IF) |
| Cleaved Casp8 (human) | Cell Signaling Technology | 9496 | 1:20(IHC) |
| Cleaved Casp8 (murine) | Cell Signaling Technology | 8592 | 1:500(IF)  1:100(IHC) |
| Cleaved Casp9 | Cell Signaling Technology | 9509 | 1:50(IF) |
| Ki67 | Leica Biosystems | PA0230 | 1:500(IF) |
| 4-Hydroxynonenal  (4-HNE) | Abcam | Ab46545 | 1:50 (IHC) |

**Supplementary Table 4.** List of commercially available antibodies used for Western blotting

| **Product** | **Manufacturer** | **Catalog No.** | **Dilution** |
| --- | --- | --- | --- |
| Casp8 (murine) | Enzo life sciences | ALX-804-447 | 1:800 |
| Cleaved Casp3 | Cell Signaling Technology | 9661 | 1:800 |
| Cleaved Casp8 | Cell Signaling Technology | 8592 | 1:800 |
| Casp9 | Cell Signaling Technology | 9504 | 1:1000 |
| CyP2E1 | Abcam | ab19140 | 1:1000 |
| Cytochrome C | BD Pharmingen | 556432 | 1:1000 |
| GAPDH | AbD Serotec | MCA4739 | 1:5000 |
| 4-Hydroxynonenal  (4-HNE) | Abcam | Ab46545 | 1:1000 |
| RIP3 | ProSci | 2283 | 1:1000 |
| TNFR1 | Santa Cruz | sc-8436 | 1:700 |
